# Effects of multimodal distribution of delays in brain network dynamics

**DOI:** 10.1186/1471-2202-16-S1-P109

**Published:** 2015-12-18

**Authors:** Spase Petkoski, Andreas Spiegler, Timothée Proix, Viktor Jirsa

**Affiliations:** 1 Aix-Marseille Université, Inserm, INS UMR_S 1106, 13005, Marseille, France; 2 Aix-Marseille Université, CNRS, ISM UMR 7287, 13288, Marseille, France; 3 Centre National de la Recherche Scientifique, Marseille, France

## 

Large-scale modeling of the brain is defined by the local oscillatory dynamics that are superimposed on an architecture based on a comprehensive map of neural connections in the brain - connectome [1]. Besides coupling strengths, time-delays due to transmissions via tracts are crucial features of a connectome. They represent a proxy of the spatial structure (the tract lengths) to the temporal dynamics. Thus, the most straightforward approach to model brain dynamics in space and time is to concatenate oscillatory nodes to a connectome-based network.

The analysis that we performed on the experimentally derived connectome suggests that the tract lengths - distances between different brain nodes, thus the time delays, follow a multimodal distribution.

Here, we investigated the conceptual implementation of multimodal distributions of discrete time delays of network links, and its effects on the mean-field dynamics. Because of the analytical tractability, the Kuramoto oscillator describes the temporal dynamics of each node, and the links between the nodes are symmetric but heterogeneous. Hence, we analyze synchronization in populations of phase oscillators [2], which have the same distribution of natural frequencies and coupling strengths, but their structure is defined solely by their different intra- and inter-population delays.

**Figure 1:**
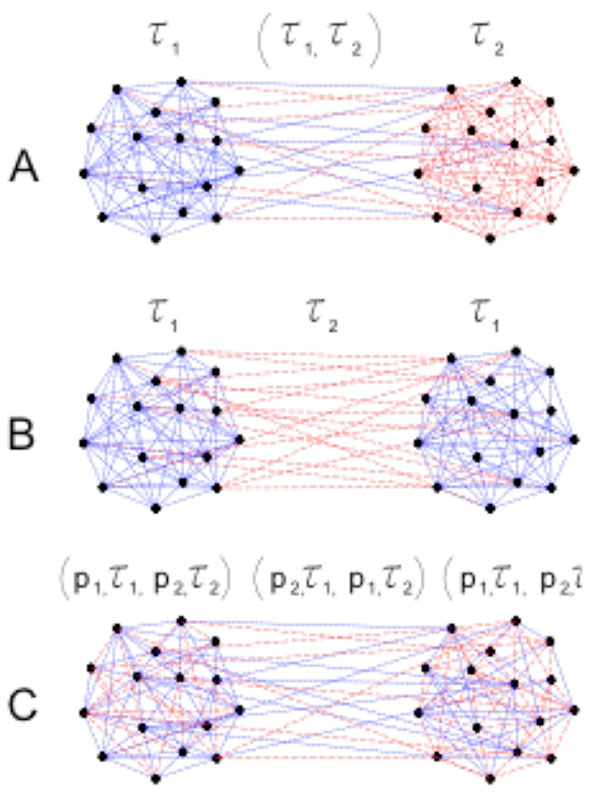
**Schematic representation of the delay-imposed structure of population of oscillators: with different inter and same intra delays in A; same inter and intra delays in B; and random distribution of the delays, in C**.

Assuming a same overall distribution of time delays, several cases are investigated: from fully random distribution, to two delays-imposed structures of subpopulations, Figure 1.

For all scenarios, mean-field dynamics are analytically obtained [3] and numerically confirmed. Moreover, boundaries and stabilities of different low-dimensional solutions are also investigated. These reveal a split of phase dynamics in different clusters, which can be phase shifted, or even non-stationary with different time-varying frequencies of synchronization and order parameters for the clusters.

In summary, the large-scale spatial organization of the brain is integrated in a network model. Using this model, we present the effects of the multimodal distribution of time delays and the structure that they impose on the network dynamics such as synchronization. Hence, we stress the role of the spatial organization of the brain that is reflected through the different time-delays between different parts of the brain in the formation of spatiotemporal dynamics.
